# Prevalence of Migraine Headache in Saudi Arabia: A Systematic Review and Meta-Analysis

**DOI:** 10.7759/cureus.37560

**Published:** 2023-04-14

**Authors:** Mohammed Faisal Albalawi, Wasan Lafi Alanazi, Hasna Saleh Albalawi, Sultan Swaulem Alghannami, Abdulmajeed F Albalawi

**Affiliations:** 1 Neurology Department, Prince Sultan Military Medical City, Riyadh, SAU; 2 Internal Medicine Department, King Faisal Specialist Hospital and Research Centre, Riyadh, SAU; 3 Emergency Medicine Department, Prince Sultan Military Medical City, Riyadh, SAU; 4 Radiology Department, Maternity and Children's Hospital, Tabuk, SAU

**Keywords:** systematic review, headache, saudi arabia, meta-analysis, migraine headache, prevalence

## Abstract

Background: Migraine is an important healthcare concern that silently affects diverse populations globally. The rising prevalence of migraine affects the quality of life of individuals, the economic burden of a nation, and work productivity. This study was designed to determine the prevalence of migraine in Saudi Arabia.

Methodology: A systematic data search was designed, and scientific data were collected from leading databases, including PubMed, The Cochrane Library, Web of Science, Ovid, and Google Scholar.

Results: Thirty-six studies, comprising 55061 study participants based on defined inclusion criteria, were statistically analyzed using StatsDirect software. The pooled proportion of migraine in Saudi Arabia among all 36 selected studies was 0.225617 (95% confidence interval (CI) = 0.172749 to 0.28326). The study was grouped into four categories: general population, students (of both genders), studies based on females only, and healthcare professionals in primary health care (PHC). The migraine pooled proportion among all four groups using random effects (DerSimonian-Laird) was 0.213822 (95% CI = 0.142888 to 0.294523), 0.205943 (95% CI = 0.127752 to 0.297076), 0.345967 (95% CI = 0.135996 to 0.593799), and 0.167068 (95% CI = 0.096429 to 0.252075), respectively.

Conclusion: The estimated pooled proportion of migraine in Saudi Arabia is 0.225617, which is comparable to or even higher than other parts of the Middle East region. Migraine has a great impact on quality of life, productivity, and economic capacity, and increases the healthcare burden. Early detection and necessary lifestyle measures are necessary to minimize this number.

## Introduction and background

Migraine is described as a frequent, chronic, episodic neurological disorder presented by diverse and variable clinical symptoms, including headache, light sensitivity, dizziness, anxiety, nausea, vomiting, etc. [1, 2]. The statistical data of the World Health Organization reported "migraine" as the third most frequent and second most damaging pathological condition, troubling more than one billion people worldwide [1]. Migraine involves the trigeminovascular system and emerges due to a disorder of sensory processing in the brain. There are many genetic and environmental factors involved in the development of this disorder, which significantly affects the quality of life (QoL) due to functional impairment leading to therapeutic interventions and is ranked as the 19th most disabling disorder globally [1, 3, 4]. The QoL is significantly deficient in patients with associated comorbidities such as diabetes mellitus, hypertension, and arthritis [4]. Moreover, females were reportedly more susceptible to migraine, especially those under 45 years of age; one in five women globally suffered from migraine, with a female-to-male ratio of 4:1 [4, 5, 6].

The prevalence of migraine is comparatively higher in Saudi Arabia than the worldwide average, with a higher rate in the female population. Saudi Arabia had approximately a 77.2% prevalence of all types of headaches, with a 25% prevalence of migraines [7]. The Global Summary of the Eastern Mediterranean Region, 1990-2016, reported that Saudi Arabia had the highest rising trend in age-standardized years lived with disability (YLD) rates of migraine and tension-type headache (TTH) [8].

The QoL of migraine patients is greatly affected by physiological and psychological involvement. The productivity and competence of migraine patients are reduced in all fields: academically, socially, occupationally, functionally, and emotionally. The financial burden is another aspect because of therapeutic demand [7]. Migraine is also associated with disability and is reportedly the eighth-highest disease linked with disability [7, 8]. There are many risk factors associated with migraines and their progressions, such as anxiety, depression, family history, gender, age, higher academic standing, obesity, stress, poor sleep hygiene, eating habits, etc. [6, 7, 9]. Caffeinated drinks, alcohol consumption, junk food, poor sleeping, and eating patterns [10] can trigger migraine attacks. The link between migraine and demographic altitude was also reported in scientific studies in Saudi Arabia [5]. Several epidemiological studies of migraine from Saudi Arabia report its predictors, symptoms, and impacts, yet no comprehensive study has reported the prevalence of migraine headaches in Saudi Arabia. We designed this systematic review and meta-analysis to identify the prevalence of migraine headaches in the Kingdom of Saudi Arabia.

## Review

Method

Data Search Strategy

Two authors of the research team were assigned to perform an identical data search. A comprehensive search of scientific articles and studies was performed. Well-known scientific databases were used, including the National Center for Biotechnology Information (NCBI)/PubMed, The Cochrane Library, Google Scholar, and Web of Science. Ovid was also used for data searches to conduct a comprehensive and systematic meta-analysis. Data were extracted with no time restriction, age, gender, or population groups to get all the possible scientific studies of migraine prevalence from Saudi Arabia.

The search keywords were defined to avoid any loss of data: ("migraine disorders"(MeSH) OR "migraine disorder" (all fields) OR migraine (all fields) OR "migraines" (all fields) OR " migraine headache" (all fields) OR "migraine headaches" OR " status migrainosus" (all fields) OR " migraine variant" (all fields) OR " migraine variants" (all fields) OR " sick headache" (all fields) OR " sick headaches" OR " abdominal migraine" (all fields) OR " cervical migraine syndrome" (all fields)) AND ("epidemiology" (MeSH Subheading) OR "epidemiology" (all fields) OR "prevalence"(all fields) OR "prevalence" (MeSH Terms) OR "prevalence" (all fields) OR "prevalence" (all fields) OR "prevalences" (all fields) OR "prevalent" (all fields) OR "prevalently" (all fields) OR "prevalent" (all fields)) AND ("cross-sectional studies" (MeSH Terms) OR ("cross-sectional" (all fields) AND "studies" (all fields)) OR "cross-sectional studies" (all fields) OR ("cross" (all fields) AND "sectional" (all fields)) OR "cross-sectional" (all fields)) NOT ("review"(Title/Abstract)), with and without Saudi Arabia.

The broad data extraction strategy was designed to avoid data loss from migraine studies in Saudi Arabia. The extracted data were critically analyzed for study selection, and selected articles were thoroughly inspected, especially the reference section, to check for data loss.

Study Selection Criteria

Inclusion and exclusion criteria: The broad inclusion criteria were established without any date or duration for data extraction. The scientific articles and studies reported the prevalence of migraine in Saudi Arabia based on all age groups and population groups of both genders. All study designs from all population facilities are included in this systematic review and meta-analysis.

Non-conclusive or incomplete studies, scientific abstract presentations, review articles, letters to the editor, case reports, and non-English articles were excluded.

Primary Outcome Measures

Gaining an accurate estimation of the prevalence rates of migraine in Saudi Arabia.

Study Selection

Studies were screened and selected by both assigned authors independently based on the inclusion criteria. Any ambiguity and difference in study selection were finalized by consensus of the research team.

Data Extraction

The risk of bias and the possibility of data loss was avoided by performing duplicate data extraction independently [11].

Methodology Statement and Data Presentation

**Figure 1:**
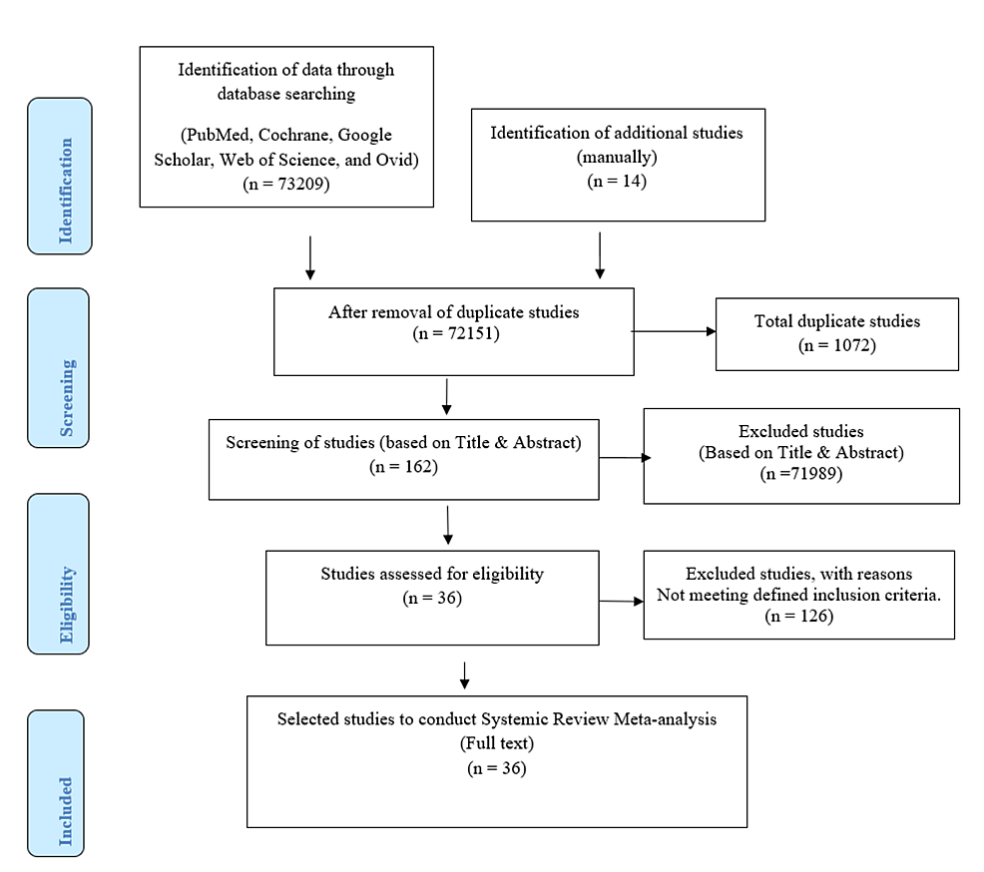
Summary of the study selection process

An overview of all identified and selected studies is presented in Table 1. Data extraction, screening, eligibility, and selection were followed by Preferred Reporting Items for Systematic Reviews and Meta-Analyses (PRISMA). This is showcased in Figure 1 [12].

**Table 1:** An overview of the selected studies FGIDs: functional gastrointestinal disorders; QoL: quality of life; CM: chronic migraine; MRI: magnetic resonance imaging; HRQL: health-related quality of life; ICHD: The International Classification of Headache Disorders; IHS: International Headache Society

S. No	First author, year, and reference no.	Study type	Duration of study	Study population	Gender	Diagnostic criteria	Total no. of study population	Migraine prevalence (%)	Additional findings
1	Al-Rajeh S, 1990 [6]	Hospital-based study	December 1983–November 1988	Population-based (hospital visit due to headache complaints)	Both	Blau definition 1984	142	27	Female preponderance aged between 11–20 years with a 4:1 female-to-male ratio
2	Abduljabbar M et al., 1996 [13]	Door-to-door survey	October 1994–March 1995 (6 months)	> 15 years	Both	IHS criteria	5891	2.6	Predominantly female
3	Al Rajeh S et al., 1997 [14]	Two-phase community-based study		All population	Both	Two-phase community-based study	16672	5	Female preponderance
4	Jabbar MA et al., 1997 [15]	A community-based door-to-door survey	_	Face-to-face questionnaire interviews, door-to-door household visits, community-based (adult population (15 years old)	Both	IHS criteria	5891	2.6	Seen more often in the professional and occupational individuals
5	Al Jumah M et al., 2002 [16]	A cross-sectional questionnaire study	1 year	School students (six–18 years)	Both	IHS criteria	1181	7.1	A sharp increase is seen in the prevalence rate of around 2% to 9% in the age group of 10 to 11 years for both genders.
6	Al-Tulaihi BA et al., 2009 [17]	A cross-sectional questionnaire study	2002–2003	16–21 years (high school students)	Both	IHS criteria	1626	7.7	Seen predominantly among female students.
7	Sabra O et al., 2015 [18]	A prospective descriptive study	_	Visited an otolaryngology clinic	Both	IHS criteria	1002	10.8	Migrainosus chief complaint group: female: 76, male: 32
8	Garah M et al., 2016 [19]	A cross-sectional study	2013-2014	University students	Female	IHS criteria	244	61.8	The most common triggering factors were 1) physical stimulation, 2) poor sleep patterns, and 3) lifestyle
9	Alwahbi MK et al. 2017 [20]	A cross-sectional observational study	2016–2017	Medical students	Both	ID Migraine™ test	270	23.7	Females: 34.1%; males: 18.6%. Severity was also seen predominantly in females.
10	Ibrahim NK et al., 2017 [21]	A cross-sectional study	2014–2015	Adults (medical students, second to sixth year)	Both	ID Migraine™ test, Numeric Pain Rating Scale (NPRS) for severity assessment	566	26.3	Severe pain cases were 41.6%. The main predictors were: 1) FGIDs, 2) family history, 3) female gender, and 4) academic pressure for the next year. The most common triggers were 1) examination stress and 2) irregular sleep.
11	Gouhar GK et al., 2018 [22]	A cross-sectional, questionnaire-based study	2016–2017	University students	Female	Migraine disability assessment scale (MIDAS)	523	44.74	The common triggering factors were stress and lack of sleep.
12	Muayqil T et al., 2018 [23]	Self-reported information collection	_	General population	Both	_	4,943	26.97	Associated with multiple comorbidities with a 1:2.9 female predominance ratio
13	Almalki ZA et al., 2018 [24]	A cross-sectional survey	During August 2017	General population	Both	IHS criteria	354	14.1	More prevalent among females, with more severe cases. females: 54%, males: 46%. Migraine with aura: 39. migraine without aura: 11
14	Khairoalsindi OA et al., 2018 [25]	An observational, cross-sectional study	February 2017	University students	Both	IHS criteria: ICHD-3 beta	558	Migraine with aura: 5.7	Probable migraine with aura: 0.5, probable migraines without aura: 7.2
								Migraine without aura: 7.5	
15	Akour A et al., 2018 [26]	A cross-sectional study	May 2017–October 2017	Medical students in the second to the sixth year (19–25 years)	Both	IHS criteria	258	5	No significant difference between genders. Common triggering factors were fatigue, minimal sleep, and the use of bright light.
16	Almesned IS et al., 2018 [27]	A cross-sectional study	December 2017–January 2018	Adults (medical students, third and fourth year)	Both	Headache Intake Questionnaire of the Cleveland Clinic, Toronto, Ontario, Canada	264	7.1	Females: 5.30%, males: 1.8%
17	Alotaibi SS et al., 2018 [28]	A cross-sectional study	23rd of November until the 21st of December 2017	General population	Both	ID Migraine screening test	450	27.3	The most common triggering factors were 1) stress: 32.5%, 2) photophobia: 63.4%. The frequency of migraine in young adults is 49.6%.
18	Desouky DE et al., 2019 [29]	A cross-sectional study	February to June 2017	University students	Female	IHS criteria	1340	32.5	Depression prevalence was significantly higher among migraine individuals.
19	AlNasser MR et al., 2019 [30]	An observational descriptive cross-sectional design	2019	Medical students 18-32 years	Both	IHS criteria	1000	15	Significantly affects QoL, academic performance, and productivity. The common triggers were 1) prolonged use of computer work, and 2) minimal sleep, caffeine, food, and exercise.
20	Rafique N et al., 2020 [31]	A case-control study	March 2019–March 2020	University students	Female	IHS criteria	1990	5.17	Poor sleep is not linked with migraine; however, stress was significantly correlated with migraine.
21	Al-Garni MA et al., 2020 [32]	Retrospective cross-sectional study	2019	Adults > 18 years visited the neurology department of the healthcare center.	Both	IHS criteria	45	46.6	CM: 35.6% episodic migraine: 11.1% females: 73.3% males: 26.7% females were more affected. The most frequent age group was 26–36 years. MRI studies showed normal results.
22	AlQarni MA et al., 2020 [33]	A descriptive cross-sectional survey	April 2018–December 2018	The general population, adults > 18 years	Both	ICHD‑III criteria	1123	12.3	Twice as common in females with insufficient sleeping habits.
23	Al Jumah M et al., 2020 [34]	A cross-sectional questionnaire-based survey	Project of the Global Campaign against Headache	The general population, adults 18–65 years	Both	ICHD-II	2316	25	Negatively linked with age > 45 years
24	Shehata SF et al., 2020 [35]	A descriptive cross-sectional study	2019–2020	University students, 18–30 years	Both	IHS criteria	421	31.2	Triggering factors: 1) irregular sleeping patterns, 2) studying hours, and 3) stress.
25	Mansour AE et al., 2021 [36]	Quantitative cross-sectional study	2020	Health college students (19–32 years)	Both	IHS criteria	456	30.7	Females had more severe migraine complaints compared to males.
26	Abukanna AMA et al., 2021 [37]	A descriptive cross-sectional study	April–July 2021	Adults, the general Saudi population, primary health care centers (PHC)	Both	HIT-6	337	36.2	Males: 19.3%, females: 80.7%, impact on HRQL: 41.2% of gender and average family monthly income are significantly associated.
27	Bamalan BA et al., 2021 [38]	A descriptive cross-sectional study	April–June 2021	The general population (adults, 18–60 years)	Both	ID-migraine scale	2058	37.2	Females: 81.1%, The most common manifestation was photophobia: 94.6%, The most frequent triggers were 1) sleep deprivation, 2) stress, and 3) anxiety
28	Kanjo M et al., 2021 [39]	A cross-sectional study	June–September 2020	Medical college students	Both	Online questionnaire	313	71.6	Female predominance
29	Alharbi, HS et al., 2021 [40]	A cross-sectional study	March and May 2021	Medical students at the university	Both	Migraine Screen Questionnaire (MSQ-5)	406	23.2	Males: 11.9%, females: 35.2%. A common triggering factor was irregular sleep
30	Alfaifi FJS et al., 2021 [41]	A cross-sectional study	February 2020–December 2020	Healthcare workers at the primary healthcare (PHC)	Both	Migraine Disability Assessment (MIDAS) test	212	20.8	Participants' health-related quality of life grades; high: 9%, moderate: 86.3%, severe: 4.7%
31	Alghamdi AA et al., 2022 [42]	A cross-sectional study	2018	Geriatric population Primary healthcare centers, adults aged 65 to 71 years	Both	Arabic Migraine Screen Questionnaire (MSQ)	75	30.7	Females (100%). Individuals were older, from rural regions, illiterate, or widows, and had a poor QoL score.
32	AlBarqi M et al., 2022 [43]	A cross-sectional questionnaire-based study	August 2021 and March 2022.	General population, adults > 18 years	Both	HARDSHIP algorithm	780	15	Probable migraine: 32.1%
33	Al-Qurashi SM, & Alsaedi MG. 2022 [44]	A cross-sectional study	2020	Primary health care physicians	Both	MIDAS score	157	12.7	Significantly higher among females
34	Ras YA et al., 2022 [45]	A cross-sectional Study	2018	≥16 years of age.	Both	ICHD‑III criteria	320	20.6	Depression had a significant association
35	Aladdin Y S et al., 2022 [46]	A questionnaire-based study	_	Students and employees of the university	Female	IHS criteria	352	38.9	Excessive consumption of coffee and caffeinated items are significantly linked
36	Alatawi AM et al., 2023 [47]	A cross-sectional study	October to November 2022	General population >18 years	Both	ID-Migraine TM questionnaire	374	64.2	Significantly linked with female gender, family history, and anxiety

Results

Study Selection Process

A total of 73209 studies were extracted after broad data extraction from scientific databases, including PubMed, Cochrane, Web of Science, Ovid, and Google Scholar. Fourteen studies were included in the extraction pool from the manual search. During the screening of articles, 1072 records were identified as duplicates. The data were screened based on the title and abstract, and 162 records were identified as eligible. Thirty-six studies were selected based on the defined inclusion criteria for this systematic review and meta-analysis; 126 records were excluded for not meeting the inclusion criteria. The PRISMA flowchart describes the complete data selection process, shown in Figure 1.

Statistical Analysis

Statistical analysis was performed using StatsDirect (version 3.0 of StatsDirect.exe; https://www.statsdirect.com/). The Miller (exact inverse Freeman-Tukey double arcsine) method was used to conduct proportion meta-analysis.

Cumulative Analysis

**Figure 2:**
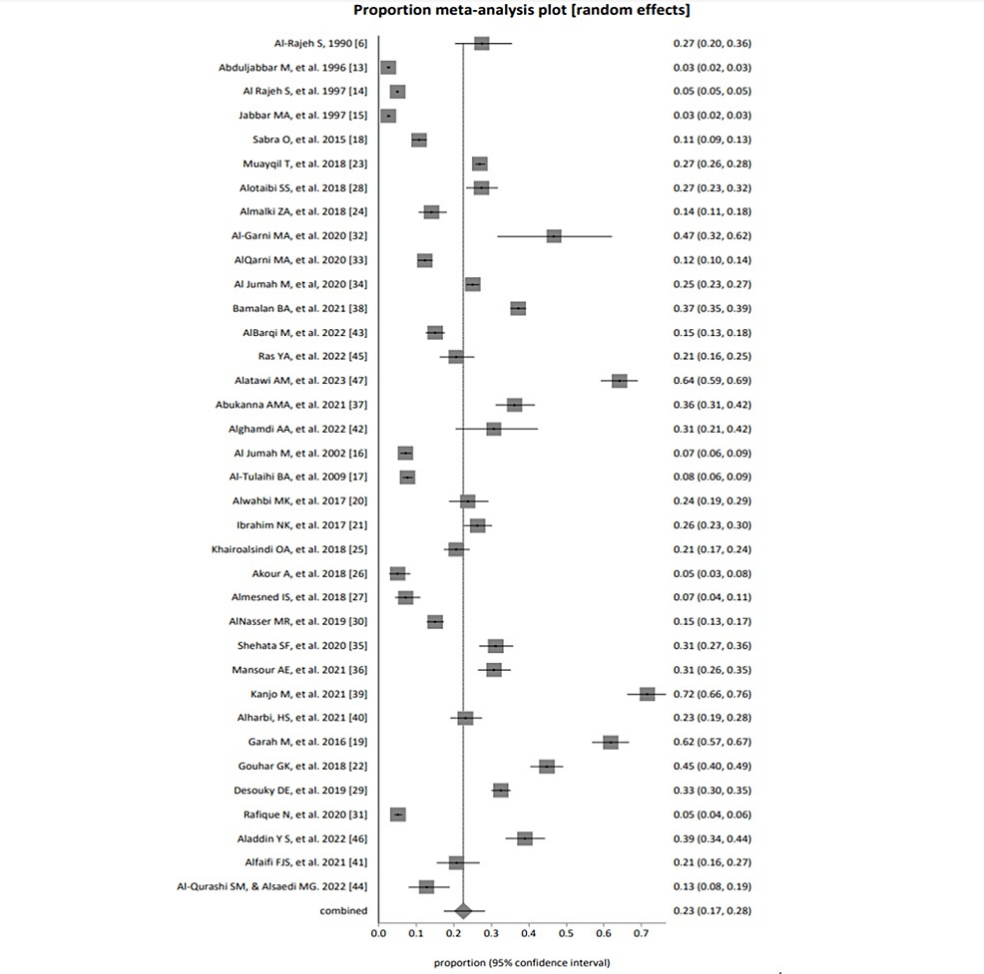
A forest plot presentation of all selected studies of migraine prevalence in Saudi Arabia [6, 13-47]

**Figure 3:**
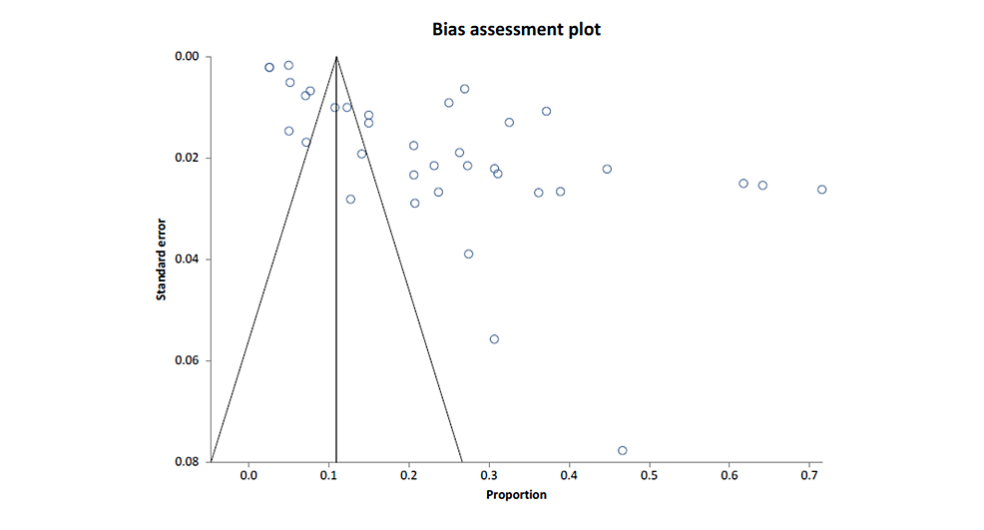
A funnel plot presentation of all selected studies of migraine prevalence in Saudi Arabia

For all selected studies, the fixed effects (inverse variance) pooled proportion = 0.109822 (95% confidence interval (CI) = 0.107205 to 0.112467); non-combinability of studies Cochran Q = 7,711.587588 (df = 35), p < 0.0001; a moment-based estimate of between-study variance = 0.160417 I2 (inconsistency) = 99.5% (95% CI = 99.5% to 99.6%); random effects (DerSimonian-Laird) pooled proportion = 0.225617 (95% CI = 0.172749 to 0.28326) and bias indicators; Begg-Mazumdar Kendall's 0.205087, p= 0.0812; Egger: bias = 13.027592 (95% CI = 9.119096 to 16.936087), p < 0.0001; Harbord: bias = 15.918419 (92.5% CI = 8.695026 to 23.141812) P = 0.0003. The forest plot presentation against the primary outcome of all selected studies was calculated and presented in Figure 2. The bias assessment plot evaluated the publication bias against the defined primary outcome of all selected studies, as shown in Figure 3.

Group Analysis

**Figure 4:**
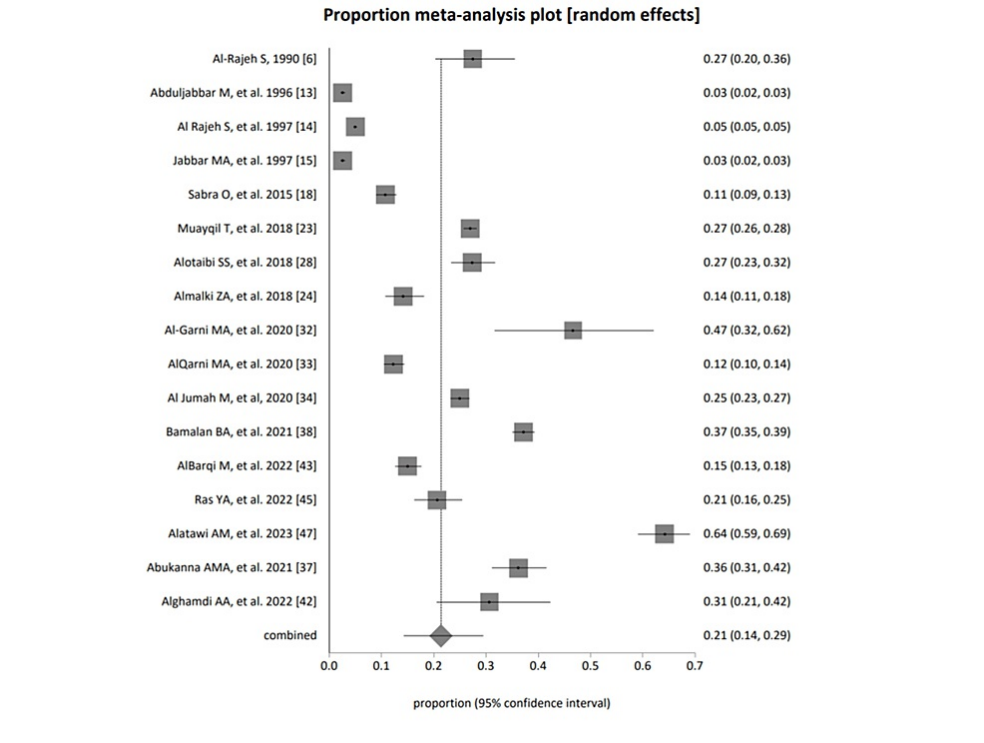
A forest plot presentation of studies based on group category; general population [6, 13-15, 18, 23-24, 28,32-34, 37-38, 42-43, 45, 47]

**Figure 5:**
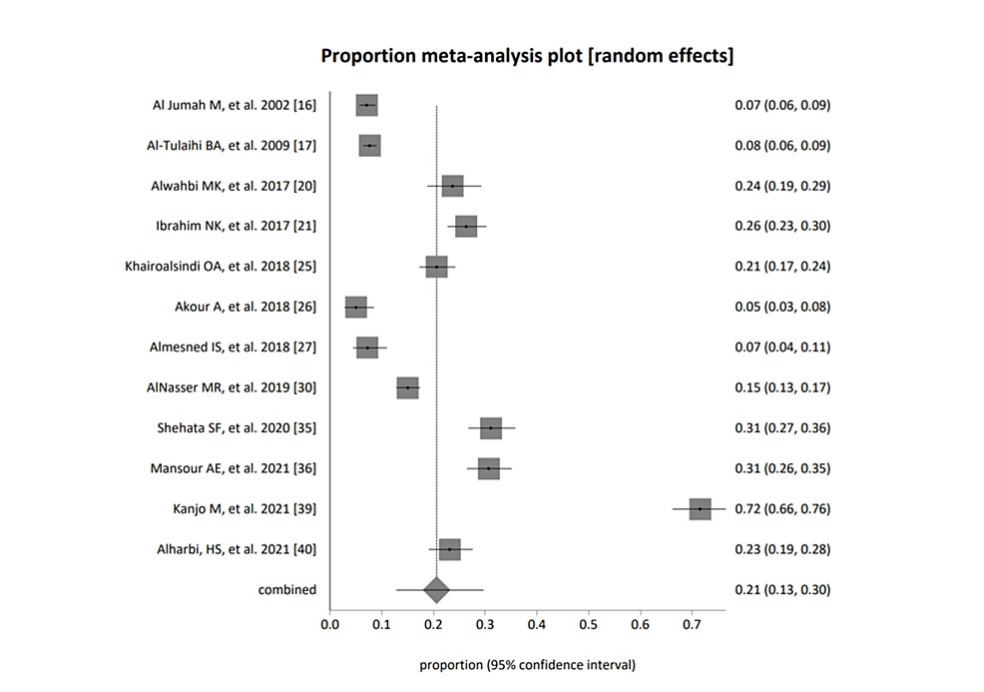
A forest plot presentation of studies based on group category; students (both genders) [16-17, 20-21,25-27, 30, 35-36, 39-40]

**Figure 6:**
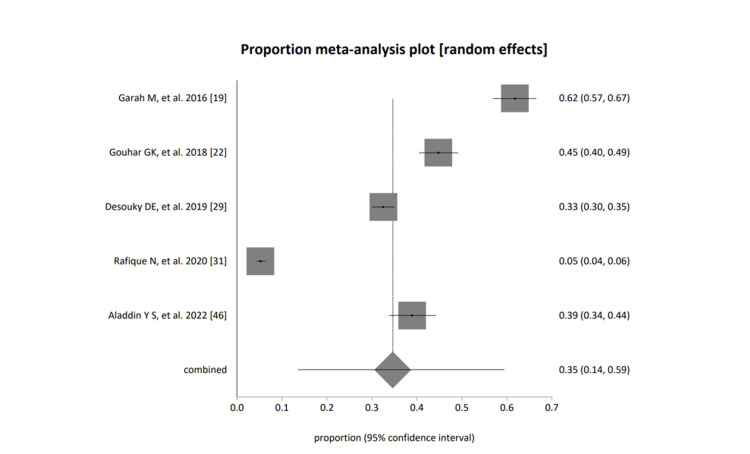
A forest plot presentation of studies based on group category; female only (students) [19, 22, 29, 31, 46]

**Figure 7:**
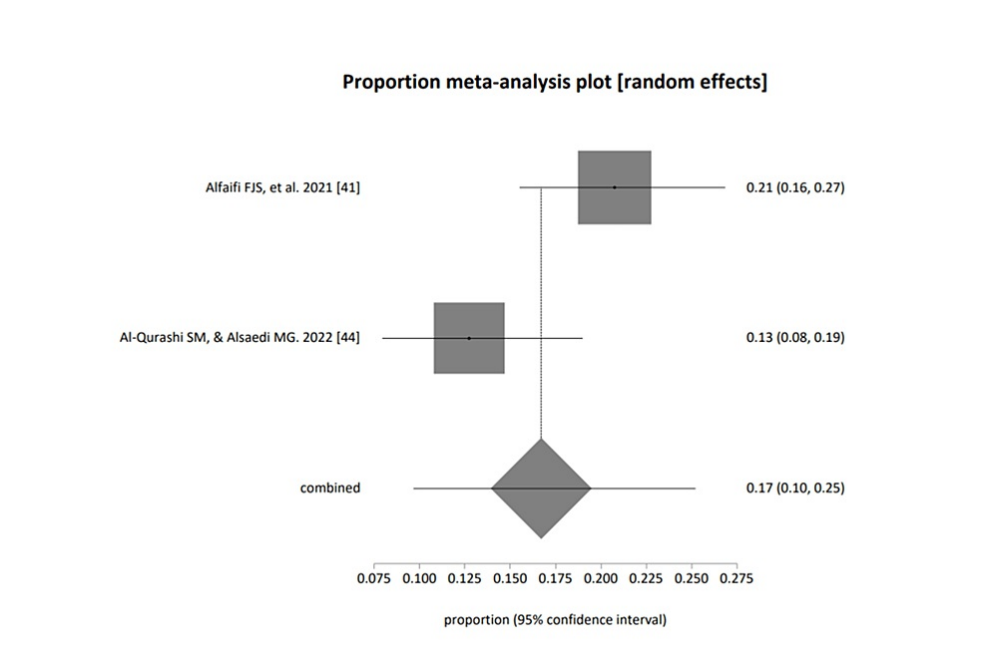
A forest plot presentation of studies based on group category; healthcare professionals (PHC) [41, 44]

**Figure 8:**
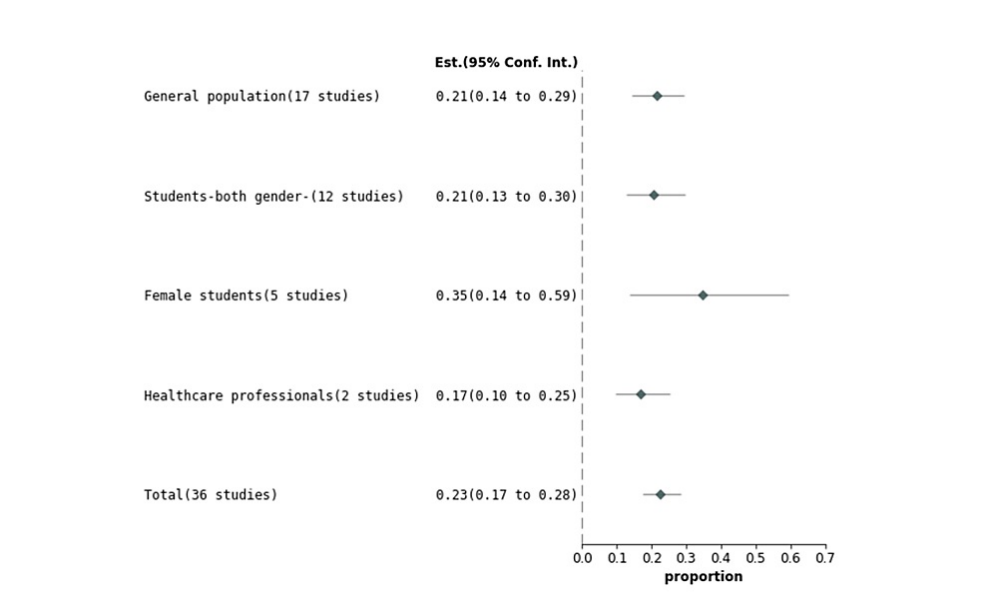
A forest plot presentation of all selected studies, cumulative and based on all groups

Studies were grouped into the general population, students (both genders), female students only, and healthcare professionals of PHC. Figures 4-7 represent the forest plot presentations of the general population, students (both genders), female students only, and healthcare professionals of PHC. The cumulative group analysis was performed and presented in Figure 8.

The fixed effects (inverse variance) pooled proportion = 0.091365 (95% CI = 0.088624 to 0.094142); non-combinability of studies Cochran Q = 5,018.090083 (df = 16), p < 0.0001, a moment-based estimate of between-study variance = 0.148071 I2 (inconsistency) = 99.7% (95% CI = 99.7% to 99.7%); random effects (DerSimonian-Laird) pooled proportion = 0.213822 (95% CI = 0.142888 to 0.294523); and bias indicators Begg-Mazumdar Kendall's 0.066667, p = 0.7413; Egger: bias = 14.199041 (95% CI = 6.810952 to 21.58713), p= 0.001; Harbord bias = 17.994527 (92.5% CI = 4.299715 to 31.689339), p= 0.0238 of the general Saudi population is represented in Figure 4.

Figure 5 represents the students (both genders), fixed effects (inverse variance), pooled proportion = 0.163133 (95% CI = 0.154725 to 0.171718), and non-combinability of studies. Cochran Q = 883.911769 (df = 11), p< 0.0001, a moment-based estimate of between-study variance = 0.135695; I2 (inconsistency) = 98.8% (95% CI = 98.6% to 98.9%); random effects (DerSimonian-Laird) pooled proportion = 0.205943 (95% CI = 0.127752 to 0.297076); bias indicators Begg-Mazumdar Kendall's 0.69697,p=0.001; Egger: bias = 14.480266 (95% CI = 5.855108 to 23.105425), p=0.0038; Harbord: bias = 17.220001 (92.5% CI = 0.7676 to 33.672403), p=0.0642.

The pooled proportion (fixed effects, (inverse variance) = 0.220154 (95% CI = 0.208272 to 0.232269); Cochran Q = 1,053.965482 (df = 4), p< 0.0001, a moment-based estimate of between-study variance = 0.324997 I2, (inconsistency) = 99.6% (95% CI = 99.6% to 99.7%); pooled proportion (random effects (DerSimonian-Laird) = 0.345967 (95% CI = 0.135996 to 0.593799); and Begg-Mazumdar Kendall's 0.2, p=0.8167; Egger: bias = 23.873393 (95% CI = 10.453376 to 37.293411), p=0.0109; Harbord: bias = 34.715364 (92.5% CI = 0.458889 to 68.97184), p=0.0728, of females only (students), shown in Figure 6.

Figure 7 represents healthcare professional groups: pooled proportion (fixed effects, (inverse variance) = 0.171584 (95% CI = 0.134569 to 0.212007); Cochran Q = 4.083815 (df = 1), p=0.0433, a moment-based estimate of between-study variance = 0.017046 I2 (inconsistency) = 75.5%; and pooled proportion (random effects, (DerSimonian-Laird) = 0.167068 (95% CI = 0.096429 to 0.252075).

The overall representation of the cumulative prevalence of all selected studies and among all four groups, including the general population, students (both genders), female students only, and healthcare professionals, were presented in Figure 8 as 0.23 (0.17 to 0.28), 0.21 (0.14 to 0.29), 0.21 (0.13 to 0.30), 0.35 (0.14 to 0.59), and 0.17 (0.10 to 0.25), respectively.

Data Findings

Thirty-six studies were selected to conduct a systematic review and meta-analysis of migraine prevalence in Saudi Arabia. All studies were extracted from well-reputed web sources and used reliable study design and methodology for migraine assessment. Table 1 summarizes the essential findings of selected studies.

The total number of study participants among all selected studies was 55061, and the pooled proportion of migraine among all selected studies using random effects (DerSimonian-Laird) was 0.225617 (95% CI = 0.172749 to 0.28326).

Selected studies were grouped into the general population, students, studies based on females only, and healthcare professionals in PHC. The pooled proportion of migraine cases among all four groups using random effects (DerSimonian-Laird) was 0.213822 (95% CI = 0.142888 to 0.294523), 0.205943 (95% CI = 0.127752 to 0.297076), 0.345967 (95% CI = 0.135996 to 0.593799), and 0.167068 (95% CI = 0.096429 to 0.252075) among the general population, students, studies based on only females, and healthcare professionals of PHC, respectively.

Discussion

The prevalence of migraine is significantly higher in Saudi Arabia than the global average and is still underestimated and neglected [34]. The Global Summary Report of the Eastern Mediterranean Region, 1990-2016, published that among 22 countries, Saudi Arabia and Libya had the highest escalation rates of age-standardized "years lived with disability (YLD)" of migraine and tension-type headache (TTH) [8]. Among all Arab countries, the migraine prevalence was reportedly high; young adults reportedly have a 12% prevalence rate [10, 48]. Despite its high and escalating prevalence, the lack of knowledge about migraine is seen not only in the general population but also among healthcare providers [1, 49]. Saudi studies also reported the significantly negative impact of migraine on quality of life, competence, and work productivity [1]. There are many reasons associated with its increased prevalence, i.e., increased urbanization, use of medicines, and high altitude [5, 34]. Despite the high prevalence of migraine documented in different studies, no study is yet designed to report the cumulative "prevalence of migraine in Saudi Arabia." As per the literature search, there is no systematic review and meta-analysis from Saudi Arabia on "migraine prevalence."

Our study reported a 0.225617 pooled proportion of migraine among the Saudi population, based on 36 selected studies and 55061 total study participants of all age groups. A systematic review based on 357 publications reported the global prevalence of migraine at 14% [50]. Our findings are also comparable to these global numbers. Iran, a Middle Eastern country, reported a 14% prevalence of migraine based on a systematic review and meta-analysis of 30 studies' outcomes [51]. Different global regions have different migraine prevalence rates: the USA reported 11.7% [52], Germany 10.6% [53], Turkey 16.4% [54], India 22.8% [55], and the UK 14.3% [56]. The UK study also reported that migraine is estimated to affect 5.85 million adult people aged between 16 and 65 years. People undergo 190,000 migraine attacks daily, which significantly affect productivity, resulting in a loss of 25 million working days [56].

Scientific evidence has reported that gender is strongly associated with migraine prevalence, its characteristics, associated symptoms, and clinical presentation [51, 57]. Among the 36 selected studies, 17 belong to the student group, of which twelve were based on both genders and five included only female students for the migraine study. In female students, the migraine prevalence was significantly high, i.e., 0.345967%. Many students support the increased prevalence of migraine in students because of educational pressure and stressful conditions [51, 57]. Our findings also support the evidence of a 0.205943 pooled proportion of migraine in student-based studies of both genders. Selected studies also support the evidence of female predominance, except for one study by Akour et al. (2018) [26], which did not find a significant difference between genders. The main migraine predictors of student-based studies were functional gastrointestinal disorders (FGIDs), family history, lifestyle, female gender, and academic pressure for the next year. The most common migraine triggers were examination stress, phonophobia, use of bright light, and irregular sleep; see Table 1. However, one study finds out that poor sleep is not linked with migraine [31]. Excessive consumption of coffee and caffeinated items is also significantly linked with migraine prevalence in students [46]. Only one study was based on school students (ages six to 18) [16] and reported a sharp increase in the prevalence rate of around 2%-9% in the age group of 10-11 years in both genders, while all the other studies of students were based on the adult age group.

The pooled proportion of migraine in the general population is 0.213822, based on 17 studies. The first study reported by Al-Rajeh S in 1990 supports a female preponderance aged between 11 and 20 years with a 4:1 female-to-male ratio [6]. This study was based on Blau's definition from 1984 [6]. Later on, all general population-based studies supported female predominance. A study by Jabbar MA et al. of 5891 general population participants found that migraine prevalence was higher among professionals, possibly due to high stress [15]. Muayqil T et al. supported the notion that migraine prevalence is associated with multiple comorbidities [23]. Stress and poor sleep were reportedly the most common triggering factors [28, 33]. Al-Garni MA et al. reported an additional fact that in migraine cases, most MRI tests showed normal results [32]. Al Jumah M et al. found a negative link between migraine and individuals over 45 years of age [34]. The severity of migraine was also evaluated in some student-based and general population-based studies and was predominantly seen among females [20, 24, 36]. Two selected studies based on a cross-sectional design evaluated migraine prevalence in healthcare professionals using the Migraine Disability Assessment (MIDAS) test, scoring a 0.167068 pooled proportion of migraine [41, 44]. As per the MIDAS grade of disability, mild disability was seen in 45 individuals, moderate in 50, and severe in 46 healthcare professionals. This study found that males may encounter mild and moderate disabilities, whereas females are affected by little to severe disabilities [41]. Migraine prevalence was higher among older age groups and more experienced female physicians [44].

This systematic review and meta-analysis is a valuable contribution to the scientific literature because of its huge sample size, which represents the overall prevalence of migraine in Saudi Arabia across all regions. The limitations of the study are that not all studies used the same study design and diagnostic criteria, which impacts heterogeneity among studies. Another limitation is that this systematic review and meta-analysis did not portray the prevalence of migraine in every region of Saudi Arabia because of the unavailability of studies.

## Conclusions

We designed this systematic review and meta-analysis to identify the prevalence of migraine headaches in the Kingdom of Saudi Arabia. The estimated pooled proportion of migraines in Saudi Arabia is 0.225617, which is alarmingly high and difficult to control. This study helps identify the necessary measures to reduce this number. These include lifestyle modifications, stress reduction, and minimal use of caffeinated drinks. However, more studies need to be conducted in all regions of Saudi Arabia to find out a more precise outcome.
